# Plants Modify Biological Processes to Ensure Survival following Carbon Depletion: A *Lolium perenne* Model

**DOI:** 10.1371/journal.pone.0012306

**Published:** 2010-08-20

**Authors:** Julia M. Lee, Puthigae Sathish, Daniel J. Donaghy, John R. Roche

**Affiliations:** 1 DairyNZ Ltd., Hamilton, New Zealand; 2 Pastoral Genomics, c/o ViaLactia Biosciences (NZ) Ltd., Auckland, New Zealand; 3 University of Tasmania, Burnie, Tasmania, Australia; USDA-ARS, United States of America

## Abstract

**Background:**

Plants, due to their immobility, have evolved mechanisms allowing them to adapt to multiple environmental and management conditions. Short-term undesirable conditions (e.g. moisture deficit, cold temperatures) generally reduce photosynthetic carbon supply while increasing soluble carbohydrate accumulation. It is not known, however, what strategies plants may use in the long-term to adapt to situations resulting in net carbon depletion (i.e. reduced photosynthetic carbon supply and carbohydrate accumulation). In addition, many transcriptomic experiments have typically been undertaken under laboratory conditions; therefore, long-term acclimation strategies that plants use in natural environments are not well understood.

**Methodology/Principal Findings:**

Perennial ryegrass (*Lolium perenne* L.) was used as a model plant to define whether plants adapt to repetitive carbon depletion and to further elucidate their long-term acclimation mechanisms. Transcriptome changes in both lamina and stubble tissues of field-grown plants with depleted carbon reserves were characterised using reverse transcription-quantitative polymerase chain reaction (RT-qPCR). The RT-qPCR data for select key genes indicated that plants reduced fructan degradation, and increased photosynthesis and fructan synthesis capacities following carbon depletion. This acclimatory response was not sufficient to prevent a reduction (*P*<0.001) in net biomass accumulation, but ensured that the plant survived.

**Conclusions:**

Adaptations of plants with depleted carbon reserves resulted in reduced post-defoliation carbon mobilization and earlier replenishment of carbon reserves, thereby ensuring survival and continued growth. These findings will help pave the way to improve plant biomass production, for either grazing livestock or biofuel purposes.

## Introduction

Plants require carbon for growth and other biological processes. In the presence of light, carbon is obtained through photosynthesis in ‘source’ leaves. Assimilated carbon is then exported as sucrose to support growth of ‘sink’ organs, such as developing leaves and roots. Sucrose is also translocated from the leaf to sink tissue for conversion into long-term carbohydrate storage compounds such as starch or fructans. These stored carbohydrates are utilised when light interception is reduced or ceases (e.g. shading, night) or when photosynthetic activity is interrupted (e.g. defoliation). The balance between carbon supply and usage is constantly changing. Plant metabolic pathways are highly complex; therefore, determining short- and long-term carbon metabolic fluxes is important in understanding the biochemistry involved and can pave the way for rational application of biotechnology in the improvement of plants.

The ability of sessile plants to continually sense and adapt to environmental and management conditions is extremely important for their survival. Modulation of gene expression plays a key role in cellular adaptation to short- or long-term changes [1], with gene expression responses to different stresses substantially advancing our understanding of the cellular strategies used for adaptation and survival. A number of common short-term stresses (e.g. water deficit, extreme temperatures, defoliation) reduce photosynthetic carbon supply [2], [3], [4], [5]. The accumulation of water-soluble carbohydrates (WSC; includes glucose, fructose, sucrose and fructans) following water deficit and exposure to low temperature are widely regarded as adaptive plant responses to ensure survival [2], [3]. However, it is not known how plants might adapt to situations resulting in net carbon depletion (i.e. reduced photosynthetic carbon supply and initial accumulation of WSC), such as frequent defoliation [4]. In addition, many transcriptomic experiments have typically been undertaken in laboratory conditions; therefore, long-term acclimation strategies that plants use in natural environments are not well understood.

Worldwide, pasture grasslands cover approximately one quarter of the total land area, providing essential feed for livestock and contributing extensively to the world economy. Perennial ryegrass (*Lolium perenne* L.) is an important C_3_ grassland species in temperate regions [6], and is the most studied perennial monocotyledonous species at the genomic, physiological and biochemical level. A wide range of well-characterised diverse germplasm exists, including both diploid and tetraploid cultivars. These factors make this species a good model for characterising plant adaptive responses to repetitive carbon depletion. Similar trait behaviour can be expected from other polycarpic perennial monocotyledons, such as *Panicum* and *Miscanthus* spp.

Perennial ryegrass is often termed a ‘3-leaf’ plant, i.e. each tiller maintains three live leaves, with the oldest leaf (first to emerge) beginning to senesce as the youngest emerges [4]. Each leaf regrowth stage is therefore defined as the time required for the production of one fully expanded leaf per tiller. In pastoral systems, it is recommended that perennial ryegrass is defoliated between the 2- and 3-leaf stages of regrowth to ensure optimal plant growth and quality. After defoliation by livestock, a ‘U-shaped’ pattern of WSC reserve depletion and replenishment occurs, with WSC reserves utilised for growth immediately after defoliation, following which replenishment begins [4], [7]. Full WSC reserve replenishment generally occurs between the 2- and 3-leaf stages; thus, successive defoliations before this point will deplete WSC reserves [4].

**Figure 1:**
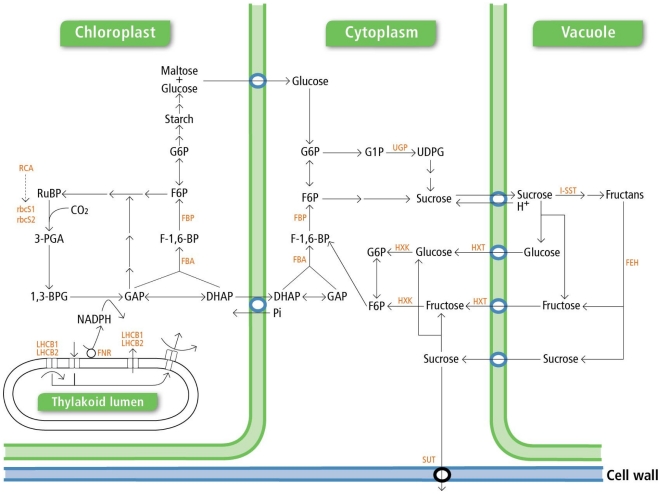
Photosynthesis and carbon metabolism and transport pathways within a plant cell. Abundance of gene transcripts encoding for the enzymes or proteins coloured red within the pathways was measured using RT-qPCR.

Perennial ryegrass was used to define whether plants adapt to repetitive carbon depletion and to discover any long-term adaptation mechanisms. Plants were either defoliated three times, each time a single new leaf had fully emerged, to deplete carbon reserves (low carbon; LC) or once following the emergence of three full new leaves (high carbon; HC). Plants were defoliated in the field (nine spatial replicates sampled over three temporal replicates for each treatment) to capture interactions between the plants and the natural environment. The experiment was timed to coincide with peak annual WSC concentrations (winter/early spring [8]), ensuring that any effects of carbon depletion were likely to be amplified during other seasons. Reverse transcription-quantitative polymerase chain reaction (RT-qPCR) was used to indicate which biological processes within the plant were adapted in response to repetitive carbon depletion. The placement of each of the profiled genes in the photosynthesis and carbon metabolism and transport pathways is indicated in Fig. 1.

## Results

### WSC Reserves and Net Biomass Accumulation

**Figure 2:**
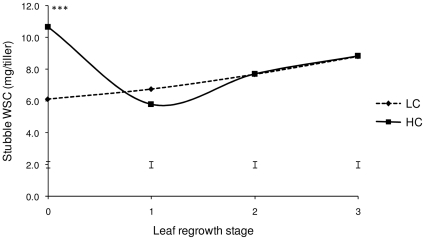
Stubble water-soluble carbohydrate (WSC) content in plants with low or high carbon reserves (LC and HC, respectively). Stubble is defined as the heterogeneous plant compartment below the defoliation height, including fully expanded leaf sheaths plus basal immature parts of expanding leaves. Significant differences between LC and HC plants are indicated by *** *P*<0.001. Error bars indicate the standard error of the difference.

**Figure 3:**
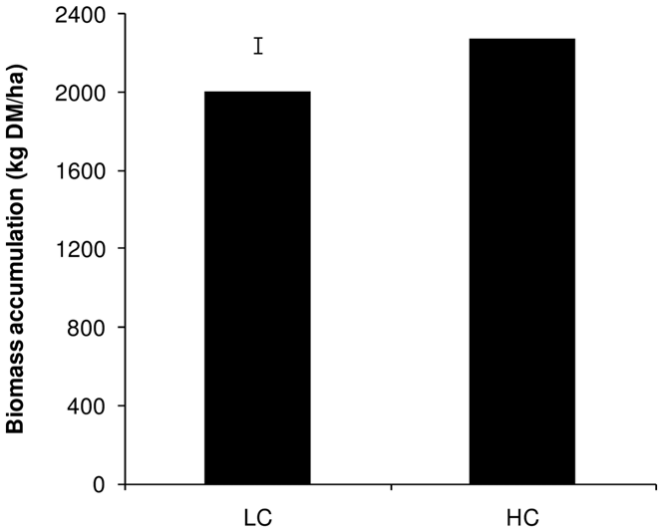
Biomass accumulation from perennial ryegrass-dominant plots containing plants with low or high carbon reserves (LC and HC, respectively). Biomass accumulation was defined as tissue that had grown above the defoliation height during the regrowth period and was measured at the 3-leaf stage of regrowth. The error bar indicates the standard error of the difference.

The stubble WSC reserves in frequently defoliated LC plants were depleted (*P*<0.001), with LC plants containing 6.1 mg WSC/tiller after defoliation compared with HC plants that contained 10.7 mg WSC/tiller (Fig. 2). There was no significant difference (*P*>0.1), however, between LC and HC plants following the appearance of the first new leaf (1-leaf stage of regrowth; Fig. 2). Net biomass accumulation at the 3-leaf stage of regrowth was reduced (*P*<0.001) in pastures containing LC plants (Fig. 3).

### Expression of Photosynthesis-related Genes

**Figure 4:**
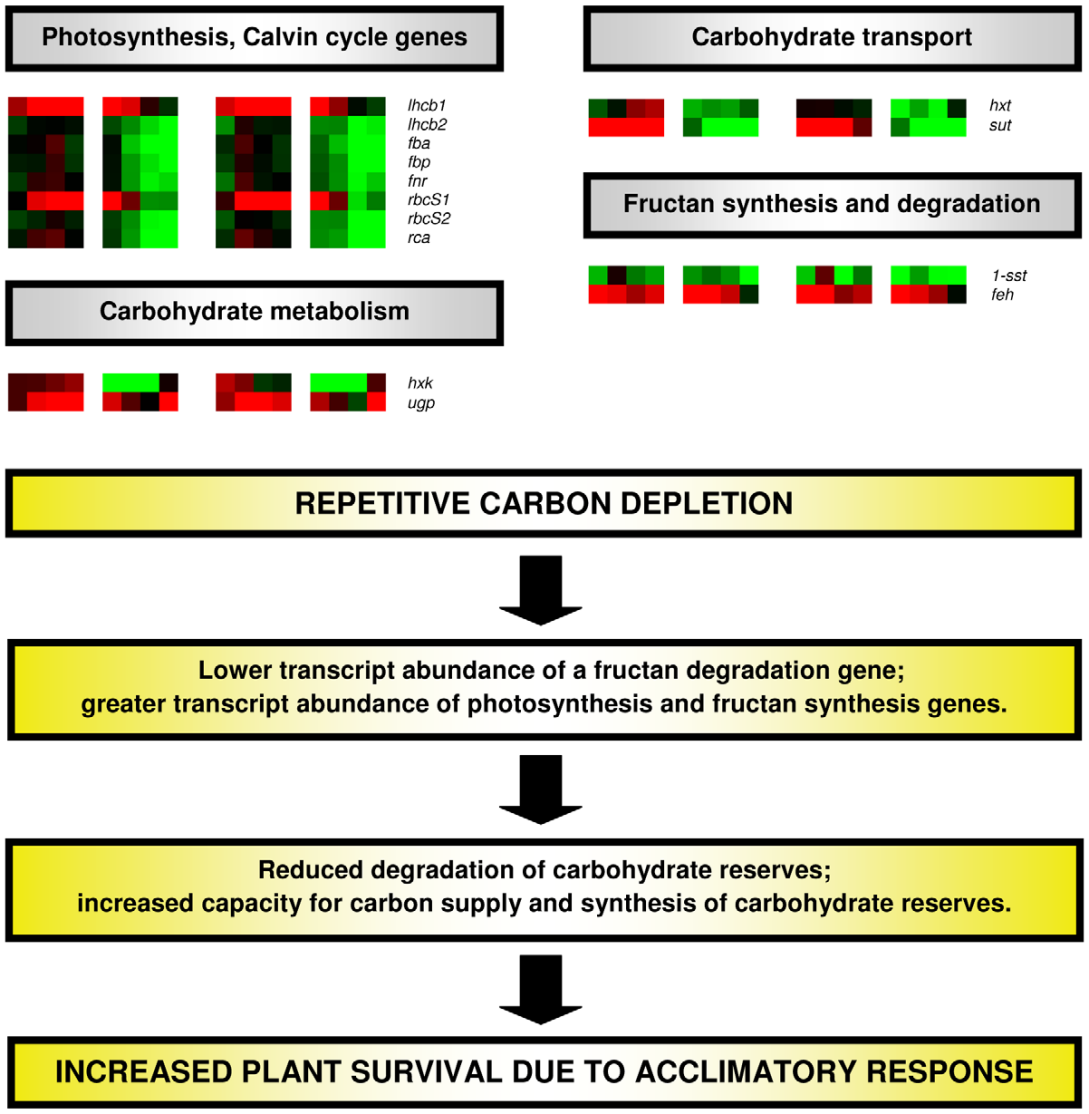
General view of gene expression in perennial ryegrass plants with low or high carbon reserves (LC and HC, respectively). Gene expression was measured on the day after defoliation, and at the 1-, 2- and 3-leaf stages of regrowth (0, 1, 2 and 3) in the lamina and stubble tissue. Values were natural log transformed and intensity in the red or green colour range indicates up- or down-regulated transcripts, respectively. Squares from left to right are LC lamina 0, 1, 2 and 3, LC stubble 0, 1, 2 and 3, HC lamina 0, 1, 2 and 3 and HC stubble 0, 1, 2 and 3. Each square represents the mean of 81 samples (3 temporal x 9 spatial x 3 RT-qPCR replicates). *lhcb1* and *lhcb2*, light harvesting chlorophyll *a*/*b* binding proteins; *fba*, fructose bisphosphate aldolase; *fbp*, fructose 1,6-bisphosphatase; *fnr*, ferredoxin NADP(H) oxidoreductase; *rbcS1* and *rbcS2*, ribulose 1,5-bisphosphate carboxylase/oxygenase (Rubisco) small subunits; *rca*, rubisco activase; *hxk*, hexokinase; *ugp*, uridine diphosphate-glucose pyrophosphorylase; *hxt*, hexose transporter; *sut*, sucrose transporter; *1-sst*, sucrose:sucrose 1-fructosyltransferase; *feh*, fructan exohydrolase.

At different stages during the regrowth period, transcripts of several genes involved in photosynthesis were more abundant in the lamina tissue of LC plants compared with HC plants (Fig. 4; normalised ratios and *P* values are presented in Table 1). These photosynthesis-related genes included the ribulose 1,5-bisphosphate carboxylase/oxygenase (Rubisco) small subunit (*rbcS2*), light harvesting chlorophyll *a/b* binding protein (*lhcb2*), Rubisco activase (*rca*), fructose bisphosphate aldolase (*fba*), fructose 1,6-bisphosphatase (*fbp*) and ferredoxin NADP(H) oxidoreductase (*fnr*) on the day after defoliation and Rubisco small subunit (*rbcS1*), *lhcb2*, *rca*, *fba*, *fbp* and *fnr* at the 2-leaf stage of regrowth.

**Table 1:** Log_10_-transformed relative transcript abundance of photosynthesis-related genes in lamina and stubble of perennial ryegrass plants that had low or high carbon reserves.

		Lamina				Stubble			
Gene	Leaf stage	LC	HC	SED	*P*	LC	HC	SED	*P*
*rbcS1*	0	−0.04	−0.01	0.054	NS	0.33	0.22	0.096	NS
	1	0.12	0.17	0.057	NS	0.02	−0.02	0.065	NS
	2	0.39	0.27	0.070	†	−0.38	−0.57	0.063	**
	3	0.34	0.27	0.075	NS	−0.41	−0.37	0.084	NS
*rbcS2*	0	−0.17	−0.23	0.039	†	−0.21	−0.35	0.069	†
	1	−0.11	−0.02	0.042	*	−0.37	−0.36	0.062	NS
	2	0.00	−0.04	0.042	NS	−0.62	−0.78	0.062	*
	3	−0.09	−0.13	0.044	NS	−0.67	−0.66	0.071	NS
*rca*	0	−0.07	−0.12	0.029	†	−0.20	−0.35	0.057	*
	1	0.04	0.06	0.030	NS	−0.40	−0.40	0.058	NS
	2	0.07	0.02	0.026	†	−0.69	−0.88	0.062	**
	3	−0.02	−0.05	0.033	NS	−0.86	−0.82	0.075	NS
*lhcb1*	0	0.09	0.05	0.085	NS	0.38	0.31	0.110	NS
	1	0.21	0.22	0.077	NS	0.11	−0.01	0.076	NS
	2	0.39	0.32	0.075	NS	−0.07	−0.14	0.088	NS
	3	0.45	0.46	0.086	NS	−0.21	−0.23	0.066	NS
*lhcb2*	0	−0.20	−0.38	0.048	***	−0.21	−0.33	0.051	*
	1	−0.05	−0.01	0.038	NS	−0.35	−0.34	0.053	NS
	2	−0.03	−0.09	0.036	†	−0.53	−0.64	0.056	†
	3	−0.06	−0.08	0.040	NS	−0.61	−0.57	0.051	NS
*fba*	0	−0.06	−0.18	0.043	**	−0.09	−0.28	0.058	***
	1	−0.01	0.04	0.028	NS	−0.48	−0.48	0.056	NS
	2	0.04	−0.03	0.039	†	−0.88	−1.07	0.060	**
	3	−0.15	−0.15	0.037	NS	−1.08	−0.96	0.090	NS
*fbp*	0	−0.09	−0.20	0.034	**	−0.08	−0.23	0.046	**
	1	−0.06	0.01	0.032	*	−0.37	−0.35	0.042	NS
	2	0.03	−0.05	0.033	*	−0.55	−0.66	0.061	†
	3	−0.12	−0.13	0.028	NS	−0.86	−0.78	0.064	NS
*fnr*	0	−0.19	−0.33	0.060	*	−0.11	−0.23	0.059	†
	1	−0.01	−0.03	0.069	NS	−0.42	−0.38	0.065	NS
	2	0.03	−0.18	0.087	*	−0.63	−0.73	0.073	NS
	3	−0.04	−0.07	0.058	NS	−0.61	−0.57	0.080	NS

LC, plants with low carbon reserves; HC, plants with high carbon reserves; SED, standard error of the difference; *rbcS1* and *rbcS2,* ribulose 1,5-bisphosphate carboxylase/oxygenase (Rubisco) small subunits; *rca*, Rubisco activase; *lhcb1* and *lhcb2*, Light harvesting chlorophyll *a*/*b* binding proteins; *fba*, fructose bisphosphate aldolase; *fbp*, fructose 1,6-bisphosphatase; *fnr*, ferredoxin NADP(H) oxidoreductase. Symbols †, *, **, *** and NS indicate significant differences between transcript abundance means at the <0.10, <0.05, <0.01 and <0.001 probability levels and lack of difference at the >0.10 probability level, respectively.

In the stubble tissue of LC plants, transcripts of several photosynthesis genes were also more abundant than in HC plants (Fig. 4; Table 1). These genes included *rbcS2*, *rca*, *lhcb2*, *fba*, *fbp* and *fnr* on the day after defoliation and *rbcs1*, *rbcs2*, *rca*, *lhcb2*, *fba* and *fbp* at the 2-leaf stage.

### Expression of Carbohydrate Metabolism and Transport-related Genes

Expression of a sucrose transporter gene (*sut*) was greater (*P*<0.05) on the day after defoliation in the lamina tissue of HC plants compared with LC plants (Fig. 4; normalised ratios and *P* values are presented in Table 2). Towards the end of the regrowth cycle, however, carbohydrate metabolism and transport gene transcripts were more abundant in lamina tissue of LC plants, with hexokinase (*hxk*), a hexose transporter (*hxt*) and uridine diphosphate glucose pyrophosphorylase (*ugp*) more abundant at the 2-leaf stage of regrowth, and *ugp* and *sut* greater at the 3-leaf stage. Transcript abundance of *hxt* was also increased (*P*<0.05) in stubble tissue of LC plants on the day after defoliation (Fig. 4; Table 2).

**Table 2:** Log_10_-transformed relative transcript abundance of carbohydrate metabolism and transport genes in lamina and stubble of perennial ryegrass plants that had low or high carbon reserves.

		Lamina				Stubble			
Gene	Leaf stage	LC	HC	SED	*P*	LC	HC	SED	*P*
*ugp*	0	0.04	0.04	0.049	NS	0.14	0.13	0.035	NS
	1	0.14	0.19	0.056	NS	0.05	0.02	0.041	NS
	2	0.31	0.18	0.060	*	−0.04	−0.10	0.051	NS
	3	0.24	0.14	0.063	†	0.41	0.42	0.035	NS
*hxk*	0	0.02	0.09	0.055	NS	−0.36	−0.37	0.033	NS
	1	−0.02	0.04	0.055	NS	−0.37	−0.42	0.042	NS
	2	0.02	−0.10	0.059	*	−0.38	−0.45	0.060	NS
	3	−0.02	−0.11	0.060	NS	0.00	0.04	0.032	NS
*hxt*	0	−0.10	−0.04	0.050	NS	−0.17	−0.26	0.040	*
	1	−0.08	−0.03	0.050	NS	−0.14	−0.19	0.045	NS
	2	0.05	−0.06	0.059	†	−0.18	−0.24	0.049	NS
	3	−0.01	−0.01	0.061	NS	−0.09	−0.04	0.027	†
*sut*	0	0.36	0.45	0.044	*	−0.11	−0.13	0.049	NS
	1	0.30	0.35	0.060	NS	−0.33	−0.31	0.047	NS
	2	0.25	0.15	0.073	NS	−0.48	−0.53	0.059	NS
	3	0.15	0.03	0.063	†	−0.36	−0.31	0.042	NS

LC, plants with low carbon reserves; HC, plants with high carbon reserves; SED, standard error of the difference; *ugp*, uridine diphosphate glucose pyrophosphorylase; *hxk*, hexokinase; *hxt*, hexose transporter; *sut*, sucrose transporter. Symbols †, * and NS indicate significant differences between transcript abundance means at the <0.10 and <0.05 probability levels and lack of difference at the >0.10 probability level, respectively.

### Expression of Fructan Degradation and Synthesis-related Genes

On the day after defoliation, transcript abundance of a gene involved in fructan synthesis (sucrose:sucrose 1-fructosyltransferase; *1-sst*) was greater (*P*<0.05) in lamina tissue of LC plants compared with HC plants (Fig. 4; normalised ratios and *P* values are presented in Table 3). This effect was also observed in stubble tissue of LC plants from the day after defoliation till the 2-leaf stage. Expression of the fructan degradation gene, fructan exohydrolase (*feh*), was less (*P*<0.01) abundant in the lamina tissue of LC plants at the 1-leaf stage of regrowth (Fig. 4; Table 3).

**Table 3:** Log_10_-transformed relative transcript abundance of fructan synthesis and degradation genes in lamina and stubble of perennial ryegrass plants that had low or high carbon reserves.

		Lamina				Stubble			
Gene	Leaf stage	LC	HC	SED	*P*	LC	HC	SED	*P*
*1-sst*	0	−0.25	−0.38	0.058	*	−0.15	−0.34	0.042	***
	1	−0.03	0.04	0.050	NS	−0.11	−0.19	0.045	†
	2	−0.25	−0.34	0.080	NS	−0.16	−0.25	0.043	*
	3	−0.49	−0.41	0.069	NS	−0.31	−0.28	0.031	NS
*feh*	0	0.14	0.17	0.031	NS	0.34	0.36	0.035	NS
	1	0.17	0.27	0.028	**	0.20	0.16	0.040	NS
	2	0.08	0.03	0.046	NS	0.14	0.11	0.039	NS
	3	0.05	0.01	0.039	NS	−0.06	−0.04	0.033	NS

LC, plants with low carbon reserves; HC, plants with high carbon reserves; SED, standard error of the difference; *1-sst*, sucrose:sucrose 1-fructosyltransferase; *feh*, fructan exohydrolase. Symbols †, *, **, *** and NS indicate significant differences between transcript abundance means at the <0.10, <0.05, <0.01 and <0.001 probability levels and lack of difference at the >0.10 probability level, respectively.

## Discussion

In the current study, frequent defoliation reduced the WSC reserves stored in LC plants after defoliation by 43%. This was consistent with previous research using a number of different pasture grasses [9], [10], [11]. After defoliation, photosynthetic energy supply is generally inadequate for growth and maintenance; therefore, WSC reserves stored in the stubble of pasture grasses are mobilized [12], [13]. The period of mobilization generally lasts until the plant has regrown ¾ to one full new leaf, after which the plant has adequate leaf area, and thus photosynthetic capacity, to meet energy requirements, as well as progressively replenish WSC reserves [4], [7]. Frequent defoliation before WSC reserves are fully replenished (i.e. before the 2-leaf stage), therefore, continually depletes the stored reserves.

In the current study, the difference in WSC reserves between LC and HC plants had dissipated by the 1-leaf stage of the subsequent regrowth period. This was the first indication of a long-term acclimatory response, suggesting modified patterns of WSC mobilization and replenishment, or alterations to the timing of each process. Smith and Stitt [14] proposed that plants acclimate to decreases in carbon supply, with declining carbon levels triggering changes in assimilation, storage and utilisation of carbon before there was a severe limitation to metabolism or growth. While such responses have been proposed based on knowledge regarding diurnal variation and the effect of extended light/dark periods on carbon content, it is logical that such responses may also be evident following defoliation frequency-induced carbon depletion.

Reduced degradation and greater synthesis of WSC are two mechanisms that plants may employ during an acclimatory response [14]. Evidence for the presence of both of these mechanisms was provided in the current study through RT-qPCR data. Transcripts of the fructan degradation gene, *feh*, were less abundant in LC plants, indicating reduced mobilization of carbon reserves, while changes in photosynthesis gene transcripts indicated an increased capacity for photosynthetic carbon supply. The increased carbon supply led to increased fructan synthesis (abundance of *1-sst*), presumably to ensure that feedback inhibition of photosynthesis did not occur, as has been previously observed in source tissues with elevated WSC content [15]. The existence of such acclimatory responses in LC plants likely explains why there was no difference in WSC content following emergence of the first new leaf.

The reduced transcript abundance of *sut* on the day after defoliation in the lamina tissue of LC plants further substantiates the hypothesis that plants with depleted carbon reserves mobilize less WSC. Following fructan degradation, fructose molecules are converted to sucrose [16], which is loaded into the phloem of carbon-exporting tissues (sources) and transported through the veins before being unloaded in carbon sink tissues [17]. The loading process is directly controlled by sucrose transporters [17]. Following defoliation, leaf sheaths and elongating leaf bases are considered to be carbon sources, while the leaf meristems enclosed in the leaf sheaths are the sinks [16], [18]. Gene expression data, however, indicates that residual lamina may also export sucrose for new lamina growth immediately after defoliation if WSC are abundant, as was the case in the HC plants with increased *sut* abundance. Rasmussen et al. [19] reported that WSC concentrations declined in the lamina of five ryegrass cultivars for 3 days post-defoliation. This suggests that WSC were mobilised from residual lamina and supports the hypothesis that sucrose may be transported for new lamina growth.

The pattern of *sut* abundance in stubble tissue throughout regrowth is similar to that observed by Berthier et al. [20], with greater transcript abundance 24 hours post-defoliation, followed by a subsequent decline. This confirms that while WSC are mobilized from the stubble to provide energy for growth after defoliation, this process is most active for only a short period immediately after defoliation [21], [22].

To our knowledge, only one other study has measured the effect of defoliation frequency on gene expression. In that study, frequent defoliation (4 times over 18 days) also reduced post-defoliation stubble WSC content [23]. During the phase of WSC replenishment, activity of two fructan synthesis enzymes and their corresponding gene transcripts were greater in plants containing less WSC. This is consistent with the current study and provides additional evidence for an acclimatory response in *L. perenne*; with plants responding to repetitive carbon depletion with increased capacity for fructan synthesis to ensure long-term carbon reserves were available for the plant later on during regrowth.

Despite the paucity of previous research regarding the effect of defoliation on gene expression, reports indicate that many photosynthesis and carbon metabolism genes were induced or repressed depending on the availability of carbohydrates in plant cells [24], [25]. Carbohydrate regulation of gene expression plays an important role in balancing the photosynthetic production of carbohydrates in source tissue with their allocation, mobilization and utilisation in sink tissue.

Although plants with depleted carbon demonstrated long-term acclimatory responses to replenish WSC reserves, biomass accumulation was still reduced, indicating that the adaptations (i.e. increased capacity for photosynthesis and WSC synthesis and decreased WSC mobilization) in LC plants were insufficient to overcome the initial repetitive carbon depletion. This is consistent with previous studies, where reductions in biomass accumulation were also evident following carbon reserve depletion in temperate pasture grasses [10], [26], [27].

A major strength of this research is that it was conducted on field-grown plants, whereas most transcriptomic research in this area has traditionally been undertaken indoors in controlled environments. Controlled environment studies do not account for interactions between weather and other confounding factors, all of which are critical to glean a more accurate picture of how plants adjust to adverse conditions [28]. The benefit of conducting field experiments was demonstrated by Sathish et al. [29], who encountered almost twice as many stress-response genes in field-grown samples than Sawbridge et al. [30] did under controlled conditions. The need for data from outdoor field studies is also highlighted by free-air carbon dioxide enrichment studies, which have not always validated data generated through controlled environment studies [31].

Previous studies report that transcript profiles of fructan metabolism genes are generally consistent with both enzymatic activity and fructan content [32], [33], [34]. This is not true for all genes, however, as post-transcriptional regulation can occur and be preponderant, at least for some genes, to transcriptional regulation. The conclusions from the current study must, therefore, be considered to be tentative. Further research is required to substantiate that changes in transcript abundance in the current study were associated with alterations to enzyme activity.

Continued progress in understanding the behaviour of the transcriptome and the metabolome in source and sink tissues is essential, especially in field-grown perennial plants that are continuously harvested for their biomass. This will improve the ability to breed plants to meet human requirements for the future with sustainable biotechnologies.

## Materials and Methods

### Plant Material and Sampling

In late June 2007, 54 plots (each 2×3 m) were established in a newly mown diploid perennial ryegrass (cv. Bronsyn) dominant sward in Hamilton, New Zealand (37°47'S 175°19'E; elevation 40 m). During the following 56-day period, plots were either defoliated three times, once each time a new leaf had fully emerged to deplete carbon reserves (LC) or once, when three new leaves had fully emerged (HC). Each treatment was replicated 27 times, comprising nine spatial replicates sampled over three temporal replicates, with the second and third temporal groups defoliated approximately 3 and 7 days after the first temporal group, respectively (see Figure S1 for exact dates). Full growth, morphological and physiological data from the study are presented by Lee et al. [35]; this paper presents a subset of that data and largely focuses on the transcriptome changes following carbon depletion.

Immediately before the final defoliation (late August), and again following the emergence of each successive full new leaf on perennial ryegrass tillers (1-, 2- and 3-leaf stages), 60 mature perennial ryegrass tillers (including roots) were harvested at random from the left-hand side of each plot for WSC analysis as described by Lee et al. [35]. Harvests were consistently performed three hours after sunrise to limit diurnal variation in WSC concentrations. After harvesting, samples were stored on ice until roots were removed from the base of the tiller and tillers were cut to their previous defoliation height (20, 40 or 60 mm) before being frozen at −20°C before freeze-drying. These samples were defined as stubble; the heterogeneous plant compartment below the defoliation height which includes fully expanded leaf sheaths plus basal immature parts of expanding leaves.

On the day after the final defoliation (late August; 0-leaf stage), and again at the 1-, 2- and 3-leaf stages, 20 individual tillers from each plot were harvested to ground level at midday for RT-qPCR analysis. Care was taken not to include dirt, floral stems or dead/diseased material in the sample. Samples were frozen immediately in liquid nitrogen, transported in dry ice and stored at −80°C. Exact collection dates are given in Figure S1. Hourly meteorological data collected during the study at a station <1 km from the experimental site are presented in Figure S2.

### Biomass Accumulation

After emergence of the third new leaf, each plot was mown to their previous defoliation height (20, 40 or 60 mm) using a rotary lawnmower as described by Lee et al. [35]. The fresh weight of this sample was recorded on a hanging scale (Salter, Victoria, Australia) suspended from a tripod in the field. Representative sub-samples (∼200 g fresh weight) were oven-dried in duplicate at 95°C to constant weight (∼48 hours) to estimate dry matter (DM) content. Biomass accumulation was calculated by multiplying the sample fresh weight by the average DM content of each plot.

### WSC Analysis

Dried samples were weighed, ground to pass through a 1 mm sieve and analysed for WSC. The WSC concentration was determined by cold extraction of plant material in a reciprocal shaker for one hour using 0.2% benzoic acid-water solution, and hydrolysation of the cold water carbohydrates extracted by 1 mol/l HCl to invert sugars. This solution was heated to 90°C, the sugar dialysed into an alkaline stream of potassium ferricyanide, and reheated to 90°C before determining WSC concentration using an autoanalyser (420 nm; Technicon Industrial Method number 302-73A; derived from the method outlined by Smith [36]). Total stubble WSC content per tiller (a better indicator for plant regrowth potential [37]) was calculated by multiplying WSC concentration by the average tiller dry weight of each sample.

### Target Gene Identification

Using Arabidopsis (*Arabidopsis thaliana* L.), Bläsing *et al.* [38] conducted three experiments to identify the 200 genes that displayed the greatest induction and the 200 genes that displayed the greatest repression following exogenous glucose supply, exogenous sucrose supply or growth in ambient CO_2_ compared with <50 ppm CO_2_. Since Arabidopsis is often used as a model for many plant species [39], an assumption was made that genes responsive to sugar in Arabidopsis, were likely to be sugar-responsive in perennial ryegrass.

The protein sequences of the sugar-responsive genes were obtained by querying the *Arabidopsis thaliana* genome database at The Institute for Genomic Research (http://www.tigr.org/tdb/e2k1/ath1/LocusNameSearch.shtml) using the gene locus names. The Arabidopsis protein sequences were TBLASTN searched against a proprietary perennial ryegrass sequence database. A hit was scored if the e value was <0.0001 and a maximum of 10 hits per Arabidopsis sequence were selected.

Additional sequences were also selected from the database based on the inclusion of keywords from photosynthesis/carbon metabolism pathways/transport systems [20], [29], [34], [40]. The orthologous ryegrass sequences identified by the above criteria were filtered based on the availability of transcriptome information, which was constructed from season-specific field-grown tissue samples using Serial Analysis of Gene Expression (SAGE) [29].

Of the genes that met these criteria, a sample of 14 genes were selected for further investigation. The genes had moderate expression profiles throughout winter and spring (5–50 copies per virtual ryegrass shoot cell as determined from SAGE data). Primer pairs were designed for the target genes using Primer3 software (http://primer3.sourceforge.net/ [41]) to amplify, whenever possible, a large portion of the 3′UTR. All primers were custom-ordered from a commercial supplier (Invitrogen, Auckland, New Zealand) and are described in Table 4.

**Table 4:** PCR primer sequences, amplicon sizes and amplification efficiency.

Gene	GenBank Accession No.	Primer sequences (5′→3′)	Region primer in	Amplicon size (bp)	Amplicon in 3′UTR (%)	Efficiency
*rbcS1*	DX922407	(F) ATATCACCTGGGTCGAGGAA	3′UTR	134	100	1.904
		(R) CCAACGGCGAATAAAGAAAC	3′UTR			(±0.013)
*rbcS2*	EC778436	(F) GAGGAGTCCGGCAAGGCATAA	coding	74	72	1.950
		(R) TATGCTTTTACATGTAGCCGGTTC	3′UTR			(±0.039)
*rca*	GO924784	(F) CCAAGAACTTCGACCCAACTG	coding	142	58	1.937
		(R) AACTTTCATGCCCAGCCATC	3′UTR			(±0.026)
*lhcb1*	GO924759, GO924785, GO924789	(F) GCCGACAACTTCATTTCTGA	3′UTR	129	100	1.933
		(R) TGAGAAATAACCACAAACAGCA	3′UTR			(±0.013)
*lhcb2*	GO924771, GO924781, GO924800	(F) GCTGACCCAGTCAACAACAA	coding	138	57	1.955
		(R) GTGCAGCTACACGTTGATCC	3′UTR			(±0.005)
*fba*	GO924786	(F) CACGTGTCGGGGTACAAGT	coding	127	81	1.886
		(R) AGTACACCGCAGGAAGGAAG	3′UTR			(±0.008)
*fbp*	GS923007	(F) TGATGCAAATCCATTTTGAAG	3′UTR	130	100	1.763
		(R) TGCTCCTCTTGGTGTCTTTG	3′UTR			(±0.005)
*fnr*	GO924778, GO924792,	(F) GCAAATGTTTCTTCCCACCT	3′UTR	122	100	1.998
		(R) AACAGCTTATCGCGCTCTTT	3′UTR			(±0.073)
*ugp*	GO924802, GO924803	(F) TCCAGTTCTCCTTCCCTGAG	3′UTR	122	100	1.955
		(R) GAAGTGCAGATGGACAGAGG	3′UTR			(±0.035)
*hxk*	GO924754	(F) ACCTTCCTTCGGCTATTCTG	3′UTR	129	100	1.877
		(R) CCTCCATCTCCATTGTTCCT	3′UTR			(±0.033)
*hxt*	GS923006	(F) TCGCTGGTGGTAATTTTGTG	3′UTR	134	100	1.953
		(R) CCAGGATTACAGCCTCACG	3′UTR			(±0.077)
*sut*	GO924755	(F) GCCTCAAGACTCCGTAGAGC	3′UTR	123	100	1.977
		(R) CAGACCACCTAGCCGGTAAT	3′UTR			(±0.028)
*1-sst*	AB288056	(F) CCAAGTTTAGCTCGTGTTGC	3′UTR	116	100	1.923
		(R) TATTGTACGATCCCCATCCA	3′UTR			(±0.040)
*feh*	DQ016297, DQ073968	(F) CCTCTGACAACATGGAGGAG	coding	139	0	1.982
		(R) AAGCCTCCATAKGCTGGTGT	coding			(±0.004)

bp, base pairs; UTR, untranslated region; *rbcS1* and *rbcS2,* ribulose 1,5-bisphosphate carboxylase/oxygenase (Rubisco) small subunits; *rca*, Rubisco activase; *lhcb1* and *lhcb2*, Light harvesting chlorophyll *a*/*b* binding proteins; *fba*, fructose bisphosphate aldolase; *fbp*, fructose 1,6-bisphosphatase; *fnr*, ferredoxin NADP(H) oxidoreductase; *ugp*, uridine diphosphate glucose pyrophosphorylase; *hxk*, hexokinase; *hxt*, hexose transporter; *sut*, sucrose transporter; *1-sst*, sucrose:sucrose 1-fructosyltransferase; *feh*, fructan exohydrolase.

### RNA Extraction and cDNA Synthesis

Frozen perennial ryegrass lamina and stubble tissues were separated and ground independently with a mortar and pestle in liquid nitrogen. DNase I-treated total RNA was extracted using the RNeasy Plant Mini Kit (Qiagen, Hilden, Germany) and mRNA isolated using Dynabeads® Oligo (dT)_25_ (Invitrogen Dynal AS, Oslo, Norway). The mRNA concentration and purity were determined using a Nanodrop ND-1000 spectrophotometer (Nanodrop Technologies Inc., Wilmington, DE, USA); each mRNA sample was assayed twice and an average value determined. Messenger RNA (10 ng) was reverse transcribed using the Transcriptor First Strand cDNA Synthesis Kit (Roche Diagnostics, Mannheim, Germany) with anchored-oligo (dT)_18_ primers in total reaction volumes of 20 µl. All procedures were carried out according to the manufacturer's protocol.

### RT-qPCR Analyses

The transcript levels of the target genes were quantified using RT-qPCR in 384-well plates with a LightCycler® 480 real-time PCR instrument (Roche Diagnostics) using the LightCycler® 480 SYBR Green I Master kit (Roche Diagnostics). Twenty-seven biological replicates (three temporal and nine spatial) for each treatment were used, and three technical RT-qPCR replicates were analysed for each biological replicate. On each plate, triplicate reactions of a no-template control, two calibrator samples (one lamina and one stubble tissue collected at the 3-leaf stage of regrowth before the experiment) and a serial dilution series for determination of a standard curve were also included. The standard curve was generated using a tenfold dilution series over three dilution points that were measured in triplicate.

Each reaction contained 5 µl of 2x SYBR Green I Master mix, 2 µl PCR-grade water, 2 µl of 100-fold diluted cDNA, and 0.5 µl of each of the 10 µM forward and reverse gene-specific primers in a final volume of 10 µl. All reaction set-ups were performed using the epMotion® 5075LH automated liquid handling system (Eppendorf, Hamburg, Germany). The reactions were incubated at 95°C for 5 minutes to activate the FastStart *Taq* DNA polymerase, followed by 45 cycles at 95°C for 10 seconds, 60°C for 10 seconds, and 72°C for 8 seconds. The specificity of the PCR reaction was confirmed with a heat dissociation protocol (from 60°C to 95°C) following the final PCR cycle. This ensured the resulting fluorescence originated from a single PCR product, and did not represent non-specific primer dimers formed during PCR or an off target amplified product. Amplification of a single product of expected size was verified by gel electrophoresis on a 1.5% agarose gel (Sigma-Aldrich, St Louis, MO, USA).

LightCycler® 480 software (version 1.5; Roche Diagnostics) was used to collect the fluorescence data. Average quantification cycles (Cq; the cycle number at which the fluorescence of a reaction rose appreciably above background fluorescence) were generated for each sample using the second derivative maxima method. Samples whose coefficient of variation (CV) were greater than 1.5% were inspected; a reaction was considered an outlier if one of the triplicate reactions deviated by more than one standard deviation from the mean, and it was subsequently excluded from the analysis. Samples were repeated if exclusion of one of the reactions still did not result in a CV<1.5%.

Previous analyses of the samples using NormFinder [42] indicated that eukaryotic elongation factor 1α and YT521-B-like protein family protein were the best two-gene combination for normalisation of RT-qPCR data [43]. Normalised ratios of the target genes were, therefore, calculated using an efficiency-corrected relative quantification formula within the LightCycler Relative Quantification Software with either a lamina or stubble tissue sample of perennial ryegrass at the 3-leaf stage of regrowth as a calibrator.

Normalised ratios were transformed (natural log) and subjected to cluster analysis using Hierarchial Clustering Explorer version 3.5 (University of Maryland; http://www.cs.umd.edu/hcil/hce/). The clusters were generated by UPGMA (average score clustering) and similarity measures assessed using Euclidean distance.

### Statistical Analyses

The normalised ratios were log_10_-transformed before statistical analysis. Repeated measurements through time were analysed using the AREPMEASURES procedure in GenStat 11.1 [44]. The statistical model included defoliation frequency, time (leaf regrowth stage) and interactions between defoliation frequency and time. Due to the large number of interactions among the defoliation treatments and time, each gene was explored using ANOVA at each timepoint separately.

## Supporting Information

Figure S1 Diagrammatical representation of defoliation events (gray boxes) and dates on which samples of perennial ryegrass leaf and stubble tissue were harvested from plants containing low or high carbon reserves (LC and HC, respectively) for RT-qPCR analyses (white boxes).
(0.03 MB DOC)

Figure S2 Hourly meteorological data collected during the study. Black bars indicate the time during which the samples for qRT-PCR analyses were collected.
(0.12 MB DOC)
