# Supplementary material for: Plants Modify Biological Processes to Ensure Survival following Carbon Depletion: A Lolium perenne Model
Source: PLoS One. 2010 Aug 20;5(8):e12306. doi: 10.1371/journal.pone.0012306 (PMC2924894; doi:10.1371/journal.pone.0012306)
Supplement: Figure S1 — Diagrammatical representation of defoliation events (gray boxes) and dates on which samples of perennial ryegrass leaf and stubble tissue were harvested from plants containing low or high carbon reserves (LC and HC, respectively) for RT-qPCR analyses (white boxes). (0.03 MB DOC) [file pone.0012306.s001.doc]

**Supporting Information Figure S1**. Diagrammatical representation of defoliation events (gray boxes) and dates on which samples of perennial ryegrass leaf and stubble tissue were harvested from plants containing low or high carbon reserves (LC and HC, respectively) for qRT-PCR analyses (white boxes).
