# Supplementary material for: Plants Modify Biological Processes to Ensure Survival following Carbon Depletion: A Lolium perenne Model
Source: PLoS One. 2010 Aug 20;5(8):e12306. doi: 10.1371/journal.pone.0012306 (PMC2924894; doi:10.1371/journal.pone.0012306)
Supplement: Figure S2 — Hourly meteorological data collected during the study. Black bars indicate the time during which the samples for qRT-PCR analyses were collected. (0.12 MB DOC) [file pone.0012306.s002.doc]

**Supporting Information Figure S2.** Hourly meteorological data collected during the study. Black bars indicate the time during which the samples for qRT-PCR analyses were collected.
